# Food advertisements on television and eating habits in adolescents: a school-based study

**DOI:** 10.11606/s1518-8787.2020054001558

**Published:** 2020-05-28

**Authors:** Leandro Dragueta Delfino, William Rodrigues Tebar, Diego Augusto Santos Silva, Fernanda Caroline Staquecini Gil, Jorge Mota, Diego Giulliano Destro Christofaro

**Affiliations:** I Universidade Estadual Paulista Faculdade de Ciências e Tecnologia Programa de Pós-graduação em Ciências da Motricidade Presidente PrudenteSP Brasil Universidade Estadual Paulista. Faculdade de Ciências e Tecnologia. Programa de Pós-graduação em Ciências da Motricidade. Presidente Prudente, SP, Brasil; II Universidade Federal de Santa Catarina Centro de Desportos Departamento de Educação Física FlorianópolisSC Brasil Universidade Federal de Santa Catarina. Centro de Desportos. Departamento de Educação Física. Florianópolis, SC, Brasil; III Universidade Estadual Paulista Faculdade de Ciências e Tecnologia Programa de Pós-graduação em Fisioterapia Presidente PrudenteSP Brasil Universidade Estadual Paulista. Faculdade de Ciências e Tecnologia. Programa de Pós-graduação em Fisioterapia. Presidente Prudente, SP, Brasil; IV Universidade do Porto Faculdade de Desporto Centro de Investigação em Actividade Física, Saúde e Lazer Porto Portugal Universidade do Porto. Faculdade de Desporto. Centro de Investigação em Actividade Física, Saúde e Lazer. Porto, Portugal; V Universidade Estadual Paulista Faculdade de Ciências e Tecnologia Departamento de Educação Física Presidente PrudenteSP Brasil Universidade Estadual Paulista. Faculdade de Ciências e Tecnologia. Departamento de Educação Física. Presidente Prudente, SP, Brasil

**Keywords:** Adolescent, Feeding Behavior, Food Publicity, Food and Nutrition Education

## Abstract

**OBJECTIVE:**

To analyze the association of television food advertisements with eating habits in Brazilian adolescents.

**METHODS:**

The sample was composed of 1,011 adolescents, aged from 10–17 years. The influence of television food advertisements on eating habits, as well as food consumption and socioeconomic variables were assessed through questionnaires. A binary logistic regression was performed to assess the magnitude of the associations, adjusted for gender, age, socioeconomic status, and parental schooling.

**RESULTS:**

Of the sample, 83.3% (n = 843) reported food consumption while watching TV. Adolescents who do not consume food while watching TV had a higher weekly consumption of fruits (3.98, SD = 2.0 *versus* 3.39, SD = 2.1) and vegetables (4.1, SD = 2.2 *versus* 3.4, SD = 2.3). Adolescents that consume food while watching TV had higher weekly consumption of fried foods (3.1, SD = 2.0 *versus* 2.3, SD = 1.7), sweets (4.1, SD = 2.1 *versus* 3.3, SD = 2.1), soft drinks (3.2, SD = 2.1 *versus* 2.2, SD = 1.9), and snacks (2.3, SD = 2.0 *versus* 1.6, SD = 1.7). For 73,8% of the sample, food advertisements induce product consumerism, most commonly sweets and fast foods. Buying or asking to buy food after seeing it on the television was associated with fried foods (OR = 1.36, 95%CI = 1.03– 1.79), sweets (OR = 1.69, 95%CI = 1.30–2.18), and snacks (OR = 1.57, 95%CI = 1.12–2.22).

**CONCLUSION:**

Food advertisements were associated with greater consumption of fried foods, sweets, and snacks in adolescents, even after adjusting for confounding factors.

## INTRODUCTION

Healthy habits in childhood tend to remain during adult life and contribute to prevent comorbidities like obesity and cardiovascular diseases^1^. However, a considerable portion of the worldwide population presents overweight or obesity, closely related to the public health problem of inappropriate nutrition^2^.

Although obesity has a multifactorial cause-genetic factors, environmental influences and lifestyle behavior^3^ the most frequently reported reasons to justify the association between nutrition and obesity are excessive consumption of energy-dense foods^4^. Unhealthy food intake is linked to impulsivity^5^ and the observed positive correlation of frequency of advertised foods on television and its consumption suggests an influence of television advertisements exposure on food choices^6^. The lack of exposure to healthy foods and the temptation to consume unhealthy foods act as barriers to healthy choices by adolescents, independent of race, gender and age^7^.

Unhealthy eating habits has been related to iron and vitamins A and C deficiencies^8^, which may lead to physical growth and intellectual activity impairment, as well as higher risk of morbidity and mortality^9^, being a public health issue even in those not obese.

A meta-analysis with over 25,000 children observed that high consumption of sugary drinks has been linked to weight gain in children^10^. Moreover, the consumption of high-energy foods increases in the presence of activities based on screens^11^. Beliefs about the consumption of unhealthy foods and health risks tend to vary according to TV exposure: more time spent watching television is associated with lower negative and higher positive beliefs about the consequences of unhealthy food consumption^12^. Another study observed that eating snacks while watching television was a predictor of high energy food consumption^13^.

Therefore, this study aimed to analyze the association of TV food advertisements with the desire to consume food, such as the weekly consumption of high-energy foods, in adolescents, even after controlling for confounding factors.

## METHODS

### Sample Selection and Inclusion Criteria

According to the Department of Education, the city of Presidente Prudente-SP has approximately 37,000 students regularly enrolled in public and private systems of which 27,860 are enrolled in elementary school and 9,105 in high school. Approximately 20% of these are private schools. Presidente Prudente is located in the southeast region of Brazil, with a population of 207,610 inhabitants and a high human development index (HDI = 0.806)^14^.

The sample of this study comprised students aged from 10 to 17 years, all regularly enrolled in schools of the city educational system (public and private). To contemplate students in the different regions (North, South, East, West, and Central), one school was chosen from each region in a randomized process. All classes of the selected schools were evaluated. If a region lacked the minimum sample required by this research, a second school was randomly selected and evaluated.

The inclusion criteria were: I) adolescents aged 10 to 17 years; II) enrolled in elementary or middle levels of the public or private educational network; and III) who returned an informed consent form, signed by one of their parents or guardians. This study was approved by the Ethics and Research Committee of the Universidade Estadual de São Paulo (Unesp).

### Sample Calculation

Sample processing considered a prevalence of use of screen time of 70%, based on a previous study^15^. As the study was performed by conglomerates, a correction of the design effect of 1.5 was applied. Coupled with possible sample losses of 20% and considering a tolerable maximum error of five percentage points, the total minimum required for the investigation was 576 subjects.

### Dependent Variables

Use of television and advertising relationship with eating habits

Food consumption by adolescents when watching television was self-reported and assessed by the following questions:

1-a) Do you ingest food when you’re watching TV? 1-Yes; 2-No.

1-b) If “Yes,” what is your favorite food to eat when watching TV?

1-Fruits; 2-Vegetables; 3-Dairy; 4-Fried food; 5-Sweets; 6-Soft drinks; 7-Snacks.

The influence of television advertisements on food consumption was assessed by the following questions:

2-a) When you are watching TV, do the food advertisements make you want to eat? 1-Yes; 2-No

2-b) If “Yes,” what kind of food do they make you want to eat?

1-Fruits; 2-Vegetables; 3-Dairy; 4-Fried food; 5-Sweets; 6-Soft drinks; 7-Snacks.

#### Anthropometric measurements

For the anthropometric assessment, the adolescents were barefoot and wearing light clothes. Body mass was obtained by a digital scale (Plenna®, São Paulo, Brazil), with a precision of 0.1 kg. Height was obtained using a portable stadiometer (Sanny®, American Medical do Brasil, São Paulo, Brazil) with a maximum extension of two meters and precision of 0.1 centimeter.

#### Food consumption

Weekly food consumption was rated by weekly frequency intake of different types of food^16^. We analyzed the consumption of fruits, vegetables, sweets, soft drinks, fried foods, and dairy (cheese, yogurt, cottage cheese, etc.). The classification for high weekly food consumption considered consumption of five or more times a week of each food.

#### Socioeconomic variables

Economic condition was determined by the “Criteria for Classification of Brazil”^17^. This instrument classify the economic condition into classes A1, A2, B1, B2, C1, C2, D, and E. After the subjects’ classification, the sample was stratified between high economic class (categories A1, A2, and B1), middle economic class (B2 and C1), and low economic class (C2, D, and E).

## Statistical Analysis

Sample characterization was presented as mean and standard deviation for the consumption or non-consumption of food during the time watching television. The odds ratio (OR), estimated through binary logistic regression, assessed the association between the dependent variable (wishing to eat food after watching television commercial) and the independent variable (frequency of weekly food consumption). The adjusted model considered the variables gender, age, socioeconomic condition, and the mother’s educational. The significance level was set at 5% and the Confidence Interval at 95% (95%CI). The statistical package used was SPSS software, version 15.0.

## RESULTS

Of the 1,011 adolescents assessed in the study, 83.3% reported food consumption while watching television. When asked their favorite food to consume, 33.2% of the sample reported eating sweets, 10.5% soft drinks, 11.1% snacks, and 6.0% fast foods. Among healthy food consumption, 10.1% of the adolescents reported fruits as their favorite food, while only 6.7% and 5.7% preferred dairy or vegetable, respectively (p ≤ 0.001).

We observed the following prevalence of food patterns in adolescents: I. 16.5% of adolescents reported no food consumption when watching TV; II. 60.9% of adolescents reported unhealthy food consumption (sweets, soft drinks, snacks, and fast foods) when watching TV; and III. 22.6% of adolescents reported healthy food consumption (fruits, dairy products, and vegetables) when watching TV (p-value ≤ 0.001). Around 20% of the sample reported food consumption five days a week while watching TV and 55% on both days of the weekend.

Table 1 shows the characteristics of the sample stratified by the consumption of food during the time watching television. When compared, the groups showed differences in age, height, and body mass. Regarding the frequency of weekly food intake, the adolescents who do not consume food while watching TV had a higher weekly consumption of fruits and vegetables, while those who consumed food while watching TV had a higher weekly consumption of fried foods, sweets, soft drinks, and snacks.

**Table 1:** Sample characteristics according to weekly frequency of food intake during time watching TV.

	Eat food when watching TV		p

	No Mean (SD)	Yes Mean (SD)	
Age	12.7 (2.4)	13.2 (2.3)	0.009
Body mass (kg)	50.1 (17.3)	50.2 (14.1)	0.977
Height (cm)	152.8 (13.6)	156.3 (12.4)	0.003
Fruits	3.98 (2.0)	3.39 (2.1)	0.001
Vegetables	4.1 (2.2)	3.4 (2.3)	0.001
Dairy	4.4 (2.5)	4.3 (2.4)	0.674
Fried food	2.3 (1.7)	3.1 (2.0)	≤ 0.001
Sweets	3.3 (2.1)	4.1 (2.1)	≤ 0.001
Soft drinks	2.2 (1.9)	3.2 (2.1)	≤ 0.001
Snack foods	1.6 (1.7)	2.3 (2.0)	≤ 0.001

SD = standard deviation

A total of 73.9% of adolescents answered that food advertisements left them wishing to eat the advertised foods and 26.1% answered negatively (p-value ≤ 0.001). The Figure shows the kinds of food they wished to eat, with a prevalence of sweets and fast food, and vegetables as the least mentioned (p-value ≤ 0.001).

**Figure:**
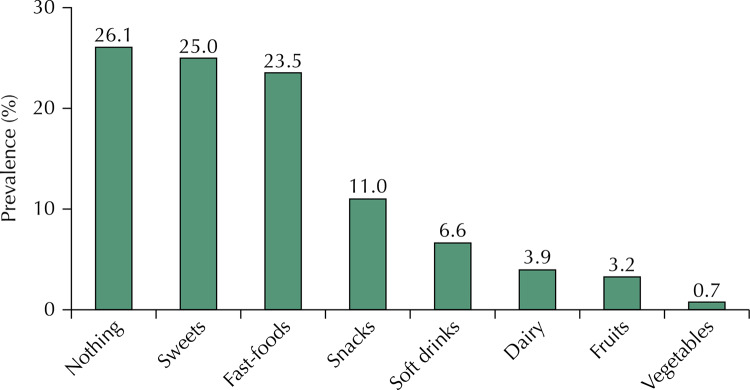
Prevalence of foods that provoke the desire to eat in adolescents after appearing in television advertisements.

Both the crude and adjusted analysis of the association between the wish to eat food after seeing it on TV advertisements and the kinds of foods, showed significant associations for the greater consumption of fried foods, sweets, soft drinks, and snacks (Table 2). Adolescents who reported wanting to eat food after seeing TV advertisements were 36% more likely to present high consumption of fried foods, 69% more likely to have a high intake of sweets, and 57% more likely to present high consumption of snacks (Table 3).

**Table 2:** Association between the influence of food product advertisements and eating habits in adolescents (variables analyzed separately in the model).

	Influence of food products advertisements				p

	Unadjusted model OR 95%CI		Adjusted model OR 95%CI		
Fruits	0.91	0.71–1.17	0.93	0.72–1.20	0.615
Vegetables	0.79	0.62–1.01	0.81	0.63–1.04	0.107
Dairy	1.02	0.79–1.32	1.05	0.81–1.36	0.832
Fried food	1.78*	1.37–2.32	1.79*	1.37–2.34	≤ 0.001
Sweets	1.72*	1.34–2.21	1.69*	1.30–2.18	≤ 0.001
Soft drinks	1.65*	1.27–2.14	1.64*	1.26–2.14	≤ 0.001
Snack foods	2.13*	1.56–2.92	2.03*	1.48–2.79	≤ 0.001

* Statistical significance at p < 0.05

Adjusted by: gender, age, socioeconomic level, and parents’ education level

**Table 3:** Association between the influence of food product advertisements and eating habits in adolescents (variables inserted simultaneously in the model).

	Influence of food products advertisements				p

	Unadjusted model OR 95%CI		Adjusted model OR 95%CI		
Fruits	0.94	0.72–1.23	0.96	0.73–1.23	0.794
Vegetables	0.82	0.63–1.08	0.84	0.64–1.10	0.205
Dairy	1.04	0.79–1.36	1.06	0.80–1.39	0.678
Fried food	1.39*	1.04–1.85	1.36*	1.03–1.79	0.018
Sweets	1.38*	1.05–1.81	1.69*	1.30–2.18	0.028
Soft drinks	1.21	0.91–1.61	1.22	1.61–2.94	0.168
Snack foods	1.66*	1.19–2.32	1.57*	1.12–2.22	0.009

* Statistical significance at p < 0.05

95%CI = 95% Confidence Interval

Adjusted by: gender, age, socioeconomic level, and parents’ education level

## DISCUSSION

This study observed that adolescents reported consuming energy-dense foods when watching television, most commonly fried foods, sweets, soft drinks, and snacks. The most desired foods to eat after seeing in TV advertisements were sweets and fast foods. Adolescents who reported wishing to eat the food after watching advertisements had higher chances of consuming fried foods, sweets and snacks-36%, 69% and 57%, respectively.

Previous studies found that fruits and vegetables intake has an inverse association with watching TV^18^, while watching TV for at least 2 hours a day leads to high energetic drink and salty snacks consumption^19^; findings corroborate those of the present study. A study warned about the observed changes in the eating habits of young Brazilians, such as increases in the intake of meat, milk, and dairy products which are rich in fats; elevation of the already excessive consumption of refined sugar and soda; and the reduction in fruits and vegetables consumption^20^. Increased caloric intake is associated with the habit of watching television, due to the high intake of foods that are dense in energy and low in nutrients^21^.

A study suggested the *theory of cultivation* as the likely cause of TV advertisement influencing young people’s food preferences^22^: advertising reduces the likelihood of individuals recognizing unhealthy behaviors, which remain unchanged^23^.

The present study revealed that the association between the habit of watching TV and unhealthy food consumption in school-aged is a reality in the country. A possible factor justifying those results is the ability of TV advertisements to stimulate food consumption in this population, even if they are not hungry^24^, inducing them to ignore satiety, and increasing their propensity for food intake^25^.

Environmental factors can also direct choices around specific products of low nutritional values and high levels of fats and sugars, while fruits and vegetables lack the same emphatical advertisements^26^. The high cost of healthier diets can also contribute to obesity among low-income groups, since the limitation of financial resources constitute an important reason why people do not eat healthy^27^. European studies support this statement: in the United Kingdom fruits and vegetables consumption were related to diet higher costs^28^; in Denmark, low-fat diets for children were associated with higher costs^29^. Thus, family eating habits influences healthy or unhealthy eating behavior in adolescents^30^.

This study advances in literature with the analysis between food requests by young people and television advertisements adjusted for possible confounding factors, like gender, age, socioeconomic condition, and the parents education level. Thus, adolescents with low socioeconomic level show low consumption of many nutritive foods like fruits, vegetables and dairy foods^31^. Consumption of unhealthy foods among adolescents was associated with mothers with more than 9 years of schooling^32^. Socioeconomic status was the only predictor associated with daily meal patterns in adolescents^33^. Concerning gender, girls reported eating vegetables and fruits more frequently than boys^34^.

The cross-sectional design, precluding cause-effect inferences, and the self-reported assessment of the variables limited this study. Food advertisements in other screen devices (computer, mobile phones, and tablets) were not assessed in the present study, since the screen time in these devices has already been associated to unhealthy eating habits in adolescents^35^. This study also lacked the assessment of types and frequency of TV food advertisements, which could help infer whether specific types of food were more frequent or more efficient in their message.

An important aspect of the study was the randomly selected sample in a developing country and the analyses controlled by confounding factors, minimizing possible biases. It also considered not only the association between screen time and weekly food consumption, but verified which kinds of food in advertisements may be associated with greater consumption. Future studies should examine food advertisements inserted between episodes of TV programs, which may contribute to better understand the intake of energy-dense foods in adolescents during television time. Another important question is whether adolescents from families with healthy habits are less susceptible to advertisements.

In conclusion, we observed a strong association between television commercials and consumption of energy-dense food. Health promotion actions should target reducing screen time and investing in advertisements for healthier foods, or at least reducing advertisements of foods rich in fat and sugar, from the early ages, since this problem affects younger populations.
